# Draft Genome Sequences of Four Bacterial Strains of Heterotrophic Alteromonas macleodii and *Marinobacter*, Isolated from a Nonaxenic Culture of Two Marine *Synechococcus* Strains

**DOI:** 10.1128/MRA.00116-21

**Published:** 2021-05-13

**Authors:** Patricia Arias-Orozco, Yunhai Yi, Oscar P. Kuipers

**Affiliations:** a Department of Molecular Genetics, Groningen Biomolecular Sciences and Biotechnology Institute, University of Groningen, Groningen, The Netherlands; b BGI Education Center, University of Chinese Academy of Sciences, Shenzhen, China; Georgia Institute of Technology

## Abstract

Species of the *Alteromonas* and *Marinobacter* genera are heterotrophic *Gammaproteobacteria* that are part of the marine microbial ecosystem. In this study, four strains were isolated from two nonaxenic *Synechococcus* cultures and were sequenced. Few studies of these two genera have been reported. Therefore, genomic data of *Alteromonadaceae* are valuable for the study of heterotroph-phototroph dynamics in marine bacterial communities.

## ANNOUNCEMENT

Marine interactions between heterotrophic and phototrophic organisms play an essential role in the nutrient cycle of marine microbial ecosystems (1). *Synechococcus* cultures are often found together with associated heterotrophic bacteria. It was previously observed that these nonaxenic cultures are more stable, possibly due to their mutually dependent relationship (2). Understanding which interactions can induce the expression of specific secondary metabolites can help in elucidating their unknown function (3).

In this work, four strains were isolated from two laboratory stocks, marine *Synechococcus* sp. strain CC9311 and *Synechococcus* sp. strain WH8102, that were a kind gift from the Department of Molecular Sciences at Macquarie University in Australia. For the isolation of the heterotrophic bacteria, both nonaxenic *Synechococcus* cultures were spread onto Difco marine agar plates and incubated at room temperature. Then, a single colony of each strain growing in Difco marine agar 2216 was selected and grown in 5 ml of marine broth 2216 without shaking and incubated for 24 h (*Alteromonas* sp.) or 48 h (*Marinobacter* sp.) at 25°C. Cells from the cultures were harvested by centrifugation at 12,000 rpm for 3 min in a Microfuge 16 centrifuge (Beckman Coulter, Woerden, The Netherlands). Genomic DNA was extracted using a GenElute bacterial genomic DNA kit (Sigma-Aldrich, Munich, Germany) according to the manufacturer’s instructions. To determine the genus of the isolated strains, we amplified and sequenced the 16S rRNA genes. According to the sequencing results, both *Synechococcus* strains coexist with one *Alteromonas* sp. and one *Marinobacter* sp.

The genomes of the four isolated strains were paired-end sequenced by the Beijing Genomics Institute European Genome Center in Denmark on a BGISEQ-500 platform. Whole-genome sequencing libraries were constructed with the MGIEasy universal DNA library prep set (MGI Tech Co., Ltd., Shenzhen, China), which is specifically designed for MGI high-throughput sequencing platform series. A total of 45 million paired-end clean reads (150 bp) were acquired, after adaptor sequences, and contamination and low-quality reads were removed from the raw reads using Trimmomatic version 0.38 (4). FastQC version 0.11.9 (5) was used to examine the quality of the reads. Subsequently, the PATRIC (6) website server was employed to perform comprehensive genome analysis. To assemble the short reads, we used Unicycler version 0.4.8 (7) integrated with SPAdes version 3.12.0 (8). The assembled draft genome sequences were evaluated with QUAST version 5.0.2 (9). Genomes were also annotated in the Prokaryotic Genome Annotation Pipeline (PGAP) (10). Default parameters were used for all software unless otherwise noted. The coverages of the four sequenced genomes were all around 350×. The assembly and annotation statistics are described in Table 1. To identify the species, we calculated the average nucleotide identity (ANI, >95% for the same species) and digital DNA-DNA hybridization (dDDH; >70% for the same species) using JSpeciesWS (11) and TYGS (12), respectively. The *Alteromonas* strain was confirmed to be A. macleodii (ANI of 98.21% and dDDH of 89.3% compared to Alteromonas macleodii ATCC 27126), while the *Marinobacter* strain could not be specified (ANI of 92.69% and dDDH of 68.2% compared to Marinobacter maroccanus). Further analysis of these genomes is under way in order to investigate their specialties as symbionts of the marine cyanobacterium *Synechococcus*.

**TABLE 1:** Genome features and accession numbers for the four *Alteromonadaceae* strains

Characteristic	Data for:			
	*Marinobacter* sp. MC3	*Marinobacter* sp. MW3	Alteromonas macleodii MC7	Alteromonas macleodii MW7
Genome size (bp)	4,732,670	4,732,670	4,771,423	4,771,559
Coverage (×)	354	352	345	352
No. of reads	11,323,466	11,316,028	11,440,920	11,459,196
No. of contigs	53	54	25	25
*N*_50_ (bp)	308,870	308,871	406,450	406,450
*L*_50_	5	6	4	4
GC content (%)	57.04	57.04	44.64	44.64
No. of genes	4,426	4,426	4,112	4,110
No. of coding sequences	4,332	4,332	4,009	4,008
No. of tRNAs	48	48	66	66
No. of rRNAs	3	3	6	5
GenBank accession no.	JAEMVF000000000	JAEMVG000000000	JAEMVH000000000	JAEMVI000000000
BioSample accession no.	SAMN17124544	SAMN17124543	SAMN17124542	SAMN17124541
SRA accession no.	SRR13297789	SRR13297790	SRR13297791	SRR13297792

### Data availability.

The genome sequences of the two *Marinobacter* strains and the two Alteromonas macleodii strains have been submitted to NCBI under BioProject PRJNA686772; see details in Table 1.
